# Grain shape is a factor affecting the stigma exsertion rate in rice

**DOI:** 10.3389/fpls.2023.1087285

**Published:** 2023-01-31

**Authors:** Quanya Tan, Songliang Chen, Zhenpeng Gan, Qimiao Lu, Zhenguang Yan, Guodong Chen, Shaojun Lin, Weifeng Yang, Jiao Zhao, Yuanyuan Ba, Haitao Zhu, Suhong Bu, Guifu Liu, Zupei Liu, Shaokui Wang, Guiquan Zhang

**Affiliations:** ^1^ Guangdong Provincial Key Laboratory of Plant Molecular Breeding, State Key Laboratory for Conservation and Utilization of Subtropical Agro-Bioresources, South China Agricultural University, Guangzhou, China; ^2^ Guangdong Laboratory for Lingnan Modern Agriculture, South China Agricultural University, Guangzhou, China

**Keywords:** grain shape, stigma shape, stigma exsertion, pleiotropic effect, outcrossing ability, rice

## Abstract

Stigma exsertion rate (SER) is an index of outcrossing ability in rice and is a key trait of male sterile lines (MSLs) in hybrid rice. In this study, it was found that the maintainer lines carrying *gs3* and *gs3*/*gw8* showed higher SER. Single-segment substitution lines (SSSLs) carrying *gs3*, *gw5*, *GW7* or *gw8* genes for grain shape and gene pyramiding lines were used to reveal the relationship between grain shape and SER. The results showed that the grain shape regulatory genes had pleiotropic effects on SER. The SERs were affected by grain shapes including grain length, grain width and the ratio of length to width (RLW) not only in low SER background, but also in high SER background. The coefficients of determination (R^2^) between grain length and SER, grain width and SER, and grain RLW and SER were 0.78, 0.72, and 0.91 respectively. The grain RLW was the most important parameter affecting SER, and a larger grain RLW was beneficial to stigma exsertion. The pyramiding line PL-*gs3*/*GW7*/*gw8* showed the largest grain RLW and the highest SER, which will be a fine breeding resource. Further research showed that the grain shape regulatory genes had pleiotropic effects on stigma shape, although the R^2^ values between grain shape and stigma shape, and stigma shape and SER were lower. Our results demonstrate that grain shape is a factor affecting SER in rice, in part by affecting stigma shape. This finding will be helpful for breeding MSLs with high SER in hybrid rice.

## Introduction

Hybrid rice has made a great contribution to maintaining the world’s major food production and food security (Yuan, 2017). The commercialization of hybrid rice depends on large-scale hybrid seed production (Qian et al., 2016). Cultivated rice is a self-pollination crop (Virmani and Athwal, 1973). The yield of hybrid seeds mainly depends on the outcrossing ability of male sterile lines (MSLs) (Virmani et al., 1982; Guo et al., 2017). The stigma exsertion in MSLs can capture more pollens from male parents, thus improving their ability of outcrossing (Marathi and Jena, 2015). Therefore, the stigma exsertion rate (SER) is an important trait for outcrossing ability in MSLs.

In the past decades, dozens of quantitative trait loci (QTLs) responsible for the SER have been identified from rice germplasm resources (Marathi and Jena, 2015; Guo et al., 2022; Liu et al., 2022). Recently, eighteen QTLs for SER from *O*. *sativa*, *O*. *glaberrima*, and *O*. *glumaepatula* were detected in the single-segment substitution lines (SSSLs) with the Huajingxian74 (HJX74) genetic background (Tan et al., 2020; Tan et al., 2021; Tan et al., 2022a). Eleven of the QTLs were used to develop the pyramiding lines with 2- to 6-QTLs in the HJX74 genetic background. The results showed that the SER can be improved with increasing QTLs in pyramiding lines. The pyramiding lines carrying 5-6 QTLs showed as high SER as wild rice (Tan et al., 2022b). The results indicate that SER is a complex trait controlled by a series of QTLs, and the high SER trait can be reconstructed by pyramiding of the QTLs in rice. However, no QTL for SER has been cloned, so the mechanism of stigma exsertion is still unclear.

SER is the result of phenotypic balance between stigma and other parts of rice spikelet (Zhou et al., 2017; Jiang et al., 2021). The relationship between SER and grain shape has become an interesting focus for researchers. Among detected QTLs, major QTLs *qES3* and *qSER8* were demonstrated to act pleiotropic effects on grain length by controlling the longitudinal axis direction of grains (Bakti and Tanaka, 2019). On the other hand, it is documented that the *GS3* gene not only determines grain length (Fan et al., 2006), but also acts a pleiotropic effect on stigma size (Takano-Kai et al., 2011; Zhou et al., 2017; Dang et al., 2020) and stigma exsertion (Miyata et al., 2007; Li et al., 2014; Zhou et al., 2017; Xu et al., 2019; Liu et al., 2022). Two cloned genes for grain width, *GW5* and *GW2*, were also reported to extend their effects on SER (Zhou et al., 2017). However, it was also reported that the *gs3* gene for long grain didn’t always result in stigma exsertion, some rice accessions with very low SER also possessed the *gs3* gene (Zhou et al., 2017; Xu et al., 2019). These results indicate that the influence of grain shape on SER is very complex, and the relationship between grain shape and SER is still not very clear.

During the process of domestication, cultivated rice has already lost the ability of natural outcrossing (Parmar et al., 1979). Wild rice has long and large stigma and long floret opening period, which provides a biological basis for outcrossing (Marathi et al., 2015). Therefore, stigma trait may be another important factor influencing SER. Some QTLs for stigma size including stigma length and stigma width were mapped (Dang et al., 2016; Zhou et al., 2017; Dang et al., 2020). Among of the QTLs, some controlled stigma size and SER (Jiang et al., 2021), while others only controlled stigma size and didn’t influence SER (Uga et al., 2003; Yan et al., 2009; Zhou et al., 2017). In addition, it was found that glume opening angle was positively correlated with SER (Kato and Namai, 1987; Uga et al., 2003; Mahalingam et al., 2013). Therefore, the relationship between stigma shape and SER is still ambiguous.

In present study, we found that the maintainer lines with long grains showed higher SERs in rice. To reveal the relationship between grain shape and SER, SSSLs carrying grain shape regulatory genes and pyramiding lines with different gene combinations were used to analyze the relationship. We show that grain shape regulatory genes have pleiotropic effects on stigma shape and SER. The grain shape is a factor affecting SER, in part by affecting stigma shape. This finding reveals the contribution of grain shape to SER, which is helpful to rebuild the outcrossing ability of MSLs in hybrid rice.

## Materials and methods

### Plant materials

The maintainer line H121B carrying four substitution segments was previously developed in the HJX74 genetic background with the *rf3* and *rf4* genes from XieqingzaoB. The maintainer line H131B was previously bred in the HJX74 genetic background with the *rf3* and *rf4* genes from XieqingzaoB and the *OsMADS50*, *gs3* and *Wx* ^t^ genes from SSSLs in the HJX74 genetic background (Dai et al., 2015). Seven SSSLs, W17-46-40-10-07-05, W23-07-06-05-02-02, W12-11-22-03-03-163, W02-08-08-08-01, W07-07-02-03-02, W23-19-06-07-19-03 and W09-38-60-07-18-04 carrying respectively *fgr*, *qBLAST-11*, *gs3*, *gs3*, *gw5*, *GW7* and *gw8* were selected from the HJX74-SSSL library (Wang et al., 2012; Zhang, 2021). The pyramiding lines carrying 2-4 QTLs for SER, 2QL-1, 2QL-5, 3QL-3, 3QL-10, 4QL-1 and 4QL-3, were previously constructed by pyramiding QTLs for SER from HJX74-SSSLs through maker assisted selection (MAS) (Tan et al., 2022b).

### Field experiment

All plant materials were planted in the experimental station, South China Agricultural University, Guangzhou (23°07′N, 113°15′E). The materials were planted in 2015-2020, two cropping seasons per year. The first cropping season (FCS) was from late February to middle July and the second cropping season (SCS) was from late July to middle November. The seeds were sown on seedbeds and the seedlings were transplanted to the paddy field as single seedlings. Field management and controlling of diseases and insect pests followed normal agricultural practices.

### Genotyping

Molecular markers were applied to detect the substitution segments from SSSLs. The length of substitution segments was measured by the method described previously (Tan et al., 2020). The target genes in substitution segments were identified using closely linked markers. The target genes were genotyped by linkage markers, functional markers, and phenotypic analysis. Genomic DNA was extracted from fresh leaf using a modified CTAB method (Murray et al., 1980). The PCR products were separated on the 6% denatured PAGE gel, and banded by the silver staining.

### Phenotyping and statistical analysis

For stigma investigation, five panicles were collected from each line during the flourishing florescence. Six mature spikelets at the upper part of each panicle were selected to carefully separate pistils from glumes and then took photos under a stereomicroscope (Leica M205FA). Rice stigma was divided into two parts, brush-shaped part (BSP) and non-brush-shaped part (NBSP). The stigma length was the sum of BSP and NBSP lengths (Takano-Kai et al., 2011). The stigma width was the maximum width of BSP (Zhou et al., 2017). Stigma length and stigma width were measured by using the software of ImageJ (https://imagej.nih.gov/ij/) described by Zhou et al. (2017). The SER was investigated following the previous method (Tan et al., 2020; Tan et al., 2021; Tan et al., 2022a). Grain traits were measured by the yield traits scorer (YTS), a rice phenotypic facility (Yang et al., 2014).

For statistical analysis, percentage data was converted to the arcsine square root. The least significance range (LSR) was used for multiple range test among multiple groups (Duncan, 1955). Student’s *t* test was used to detect the difference between two groups. The correlation between traits was analyzed by regression correlation. MapChart2.3 (https://www.wur.nl/en/show/Mapchart.htm) and OriginPro 9.0 (https://www.originlab.com) were used to make figures.

## Results

### Long grain maintainer lines showed higher SER

**Figure 1:**
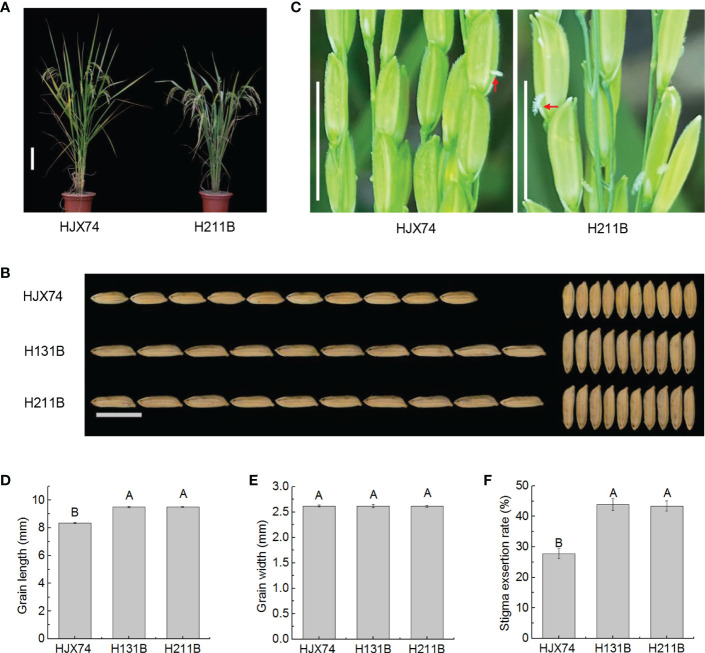
Grain shape and stigma exsertion rate (SER) in maintainer line H211B. **(A)**, Plant type of HJX74 and H211B. Scale bar, 15 cm. **(B)**, Grain shape in HJX74, H131B and H211B. Scale bar, 1 cm. **(C)**, SER in HJX74 and H211B. Scale bar, 1 cm. The arrows point the exserted stigmas. Grain length **(D)**, grain width **(E)**, and SER **(F)** in HJX74, H131B and H211B. Data are shown as mean ± S.E. of two cropping seasons. Capital letters indicate significant differences at the 0.01 level.

The maintainer line H131B previously developed in the HJX74 genetic background was improved by pyramiding two target genes on the substitution segments of HJX74-SSSLs. The new maintainer line H211B carrying seven target genes on substitution segments in the HJX74 genetic background was developed (**Supplementary Figure 1** and **Supplementary Table 1**). Phenotype investigation showed that the traits controlled by target genes have been significantly improved, while other traits had no significant difference (**Supplementary Table 2**). Both H131B and H211B carried the *gs3* gene. Compared with HJX74, H131B and H211B showed significant differences in grain length, but no significant differences in grain width. The grain length of HJX74 was 8.36 mm, while that of H131B and H211B increased to 9.50 mm and 9.51 mm respectively (**Figures 1A, B, D, E** and **Supplementary Table 2**). Interestingly, the SERs of H131B and H211B were also significantly different from HJX74. The SER of HJX74 was 27.8%, while that of H131B and H211B increased to 43.9% and 43.4% respectively (**Figures 1C, F** and **Supplementary Table 2**). These results showed that the long grain trait controlled by *gs3* increased the SERs of maintainer lines H131B and H211B.

**Figure 2:**
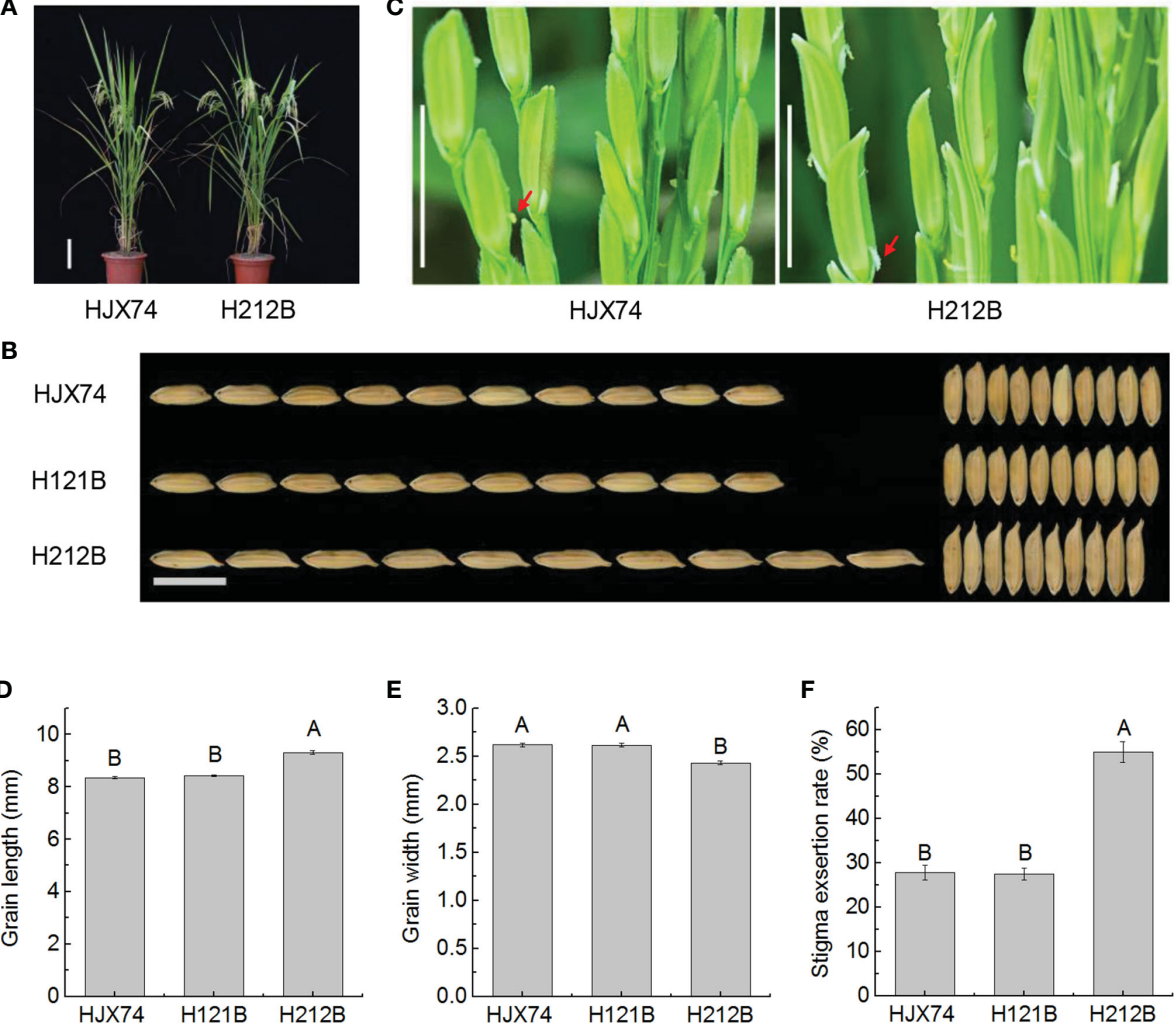
Grain shape and stigma exsertion rate (SER) in maintainer line H212B. **(A)**, Plant type of HJX74 and H212B. Scale bar, 15 cm. **(B)**, Grain shape in HJX74, H121B and H212B. Scale bar, 1 cm. **(C)**, SER in HJX74 and H212B. Scale bar, 1 cm. The arrows point the exserted stigmas. Grain length **(D)**, grain width **(E)**, and SER **(F)** in HJX74, H121B and H212B. Data are shown as mean ± S.E. of two cropping seasons. Capital letters indicate significant differences at the 0.01 level.

The maintainer line H121B previously developed in the HJX74 genetic background was improved by pyramiding *gs3* and *gw8* genes on substitution segments of HJX74-SSSLs. The new maintainer line H212B carrying *gs3*and *gw8* genes in the HJX74 genetic background was developed (**Supplementary Figure 2** and **Supplementary Table 3**). Phenotype investigation showed that the traits controlled by target genes have been significantly improved, while other traits had no significant differences (**Supplementary Table 4**). Compared with HJX74 and H121B, H212B carrying *gs3* and *gw8* genes had significant differences in grain length and grain width. In grain length, HJX74 and H121B were 8.36 mm and 8.42 mm respectively, while H212B increased to 9.31 mm. In grain width, HJX74 and H121B were both 2.62 mm, while H212B reduced to 2.43 mm (**Figures 2A, B, D, E** and **Supplementary Table 4**). Interestingly, the SER of H212B was also significantly different from that of HJX74 and H121B. SERs of HJX74 and H121B were 27.8% and 27.4% respectively, while SER of H212B increased to 55.0% (**Figures 2C, F** and **Supplementary Table 4**). Compared with H131B and H211B carrying *gs3* (**Figure 1** and **Supplementary Table 2**), H212B with *gs3* and *gw8* had slender grain and high SER.

### Pleiotropic effects on SER of the genes controlling grain shape in SSSLs

**Figure 3:**
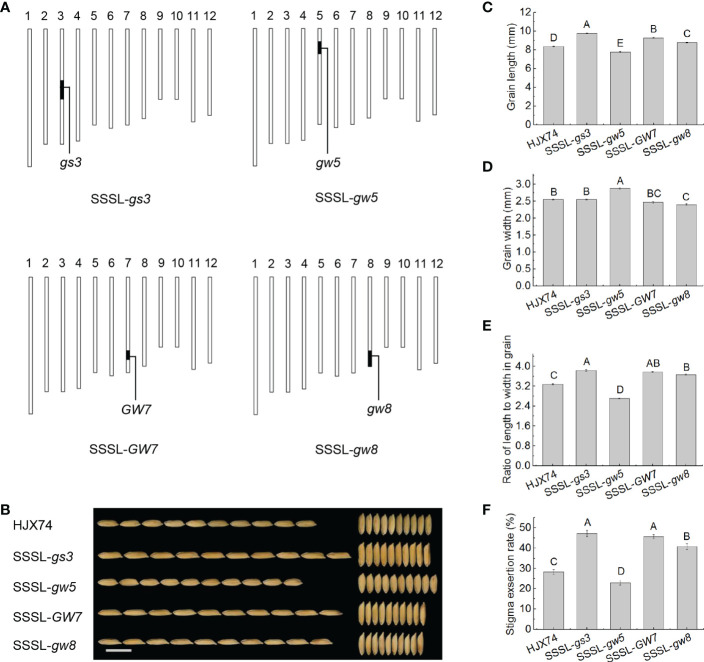
Grain shape and SER in the SSSLs carrying a gene for grain shape. **(A)**, Graphical genotypes of the SSSLs. The vertical bars are a graphical representation of chromosomes. The black bars represent substitution segments containing target genes for grain shape, and the white regions represent the HJX74 genetic background. **(B)**, Appearance of the grains in HJX74 and SSSLs. Scale bar, 1 cm. Grain length **(C)**, grain width **(D)**, grain RLW **(E)**, and SER **(F)** in HJX74 and SSSLs. Data are shown as mean ± S.E. of two cropping seasons. Capital letters indicate significant differences at the 0.01 level. SSSL, single-segment substitution line. SER, stigma exsertion rate. RLW, ratio of length to width.

In order to confirm the finding that the trait of slender grain led to higher SER, four SSSLs carrying a grain shape regulatory gene, *gs3*, *gw5*, *GW7* or *gw8*, on the substitution segments in the HJX74 genetic background were used to analyze the relationship between grain shape and SER (**Figure 3A** and **Supplementary Table 5**). The grain of HJX74 was 8.38 mm long and 2.55 mm wide. SSSL-*gs3*, SSSL-*gw5*, SSSL-*GW7* and SSSL-*gw8* had grain lengths of 9.77 mm, 7.79 mm, 9.29 mm and 8.79 mm, and grain widths of 2.55 mm, 2.88 mm, 2.47 mm and 2.40 mm, respectively. In the ratio of length to width (RLW) in grains, HJX74 was 3.29, while SSSL-*gs3*, SSSL-*GW7* and SSSL-*gw8* increased to 3.83, 3.77 and 3.65 respectively, and SSSL-*gw5* decreased to 2.71 (**Figures 3B–E** and **Supplementary Table 6**). Correspondingly, the SER of HJX74 was 28.3%, while that of SSSL-*gs3*, SSSL-*GW7* and SSSL-*gw8* increased to 47.4%, 45.6% and 40.8% respectively, and that of SSSL-*gw5* decreased to 22.8% (**Figures 3E, F** and **Supplementary Table 6**). These results showed that the genes of *gs3*, *gw5*, *GW7* and *gw8* had significant effects on grain shape and SER in the SSSLs, indicating that the genes controlling grain shape had significant pleiotropic effects on SER.

### Effects of the grain shape controlled by different gene combinations of *gs3*, *gw5*, *GW7* and *gw8* on SER in pyramiding lines

To further evaluate the effect of grain shape on SER, four SSSLs, SSSL-*gs3*, SSSL-*gw5*, SSSL-*GW7* and SSSL-*gw8*, were used to develop a series of pyramiding lines. A total of 11 pyramiding lines were developed through MAS, including six 2-gene pyramiding lines, four 3-gene pyramiding lines, and one 4-gene pyramiding line (**Supplementary Table 7**).

**Figure 4:**
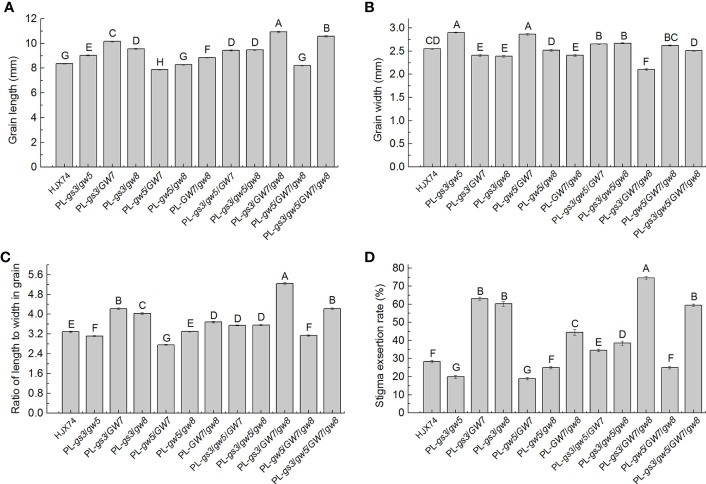
Grain shape and SER in pyramiding lines carrying genes for grain shape. Grain length **(A)**, grain width **(B)**, grain RLW **(C)**, and SER **(D)** in HJX74 and pyramiding lines carrying genes for grain shape. Data are shown as mean ± S.E. of two cropping seasons. Capital letters indicate significant differences at the 0.01 level. SER, stigma exsertion rate. PL, pyramiding line. RLW, ratio of length to width.

In six 2-gene pyramiding lines, PL-*gs3*/*gw5*, PL-*gs3*/*GW7*, PL-*gs3*/*gw8*, PL-*gw5*/*GW7*, PL-*gw5*/*gw8* and PL-*GW7*/*gw8*, grain lengths were 9.04 mm, 10.17 mm, 9.57 mm, 7.88 mm, 8.29 mm and 8.85 mm, and grain widths were 2.90 mm, 2.41 mm, 2.39 mm, 2.86 mm, 2.52 mm and 2.41 mm, respectively, which differed from 8.38 mm long and 2.55 mm wide of the HJX74 grain (**Figures 4A, B** and **Supplementary Table 8**). Compared with the grain RLW of HJX74 3.29, grain RLWs of PL-*gs3*/*gw5*, PL-*gs3*/*GW7*, PL-*gs3*/*gw8*, PL-*gw5*/*GW7*, PL-*gw5*/*gw8* and PL-*GW7*/*gw8* were 3.12, 4.22, 4.01, 2.75, 3.29 and 3.67 respectively. Correspondingly, SERs of PL-*gs3*/*gw5*, PL-*gs3*/*GW7*, PL-*gs3*/*gw8*, PL-*gw5*/*GW7*, PL-*gw5*/*gw8* and PL-*GW7*/*gw8* were 20.0%, 63.1%, 60.3%, 19.0%, 25.0% and 44.5% respectively, with 28.3% SER of HJX74 as control (**Figures 4C, D** and **Supplementary Table 8**). In five pyramiding lines with multiple genes for grain shape, PL-*gs3*/*gw5*/*GW7*, PL-*gs3*/*gw5*/*gw8*, PL-*gs3*/*GW7*/*gw8*, PL-*gw5*/*GW7*/*gw8* and PL-*gs3*/*gw5*/*GW7*/*gw8*, grain lengths were 9.43 mm, 9.49 mm, 10.93 mm, 8.22 mm and 10.59 mm, grain widths were 2.65 mm, 2.67 mm, 2.10 mm, 2.62 mm and 2.51 mm, and grain RLWs were 3.55, 3.55, 5.21, 3.14 and 4.21, respectively. SERs of the five pyramiding lines were 34.6%, 38.5%, 74.6%, 25.0% and 59.5% respectively (**Figures 4A–D** and **Supplementary Table 8**). These results showed that larger grain RLWs led to higher SERs in the pyramiding lines. Four pyramiding lines, PL-*gs3*/*GW7*, PL-*gs3*/*gw8*, PL-*gs3*/*GW7*/*gw8*, and PL-*gs3*/*gw5*/*GW7*/*gw8*, had larger than 4.0 of grain RLWs and higher than 50.0% of SERs. The pyramiding line PL-*gs3*/*GW7*/*gw8* showed the largest grain RLW and the highest SER.

### Effects of grain shape on SER in high SER background

To investigate the effect of grain shape on SER at high SER level, the *gs3* gene was introduced into the pyramiding lines carrying multiple SER-QTLs, 2-QTL pyramiding lines (2QLs), 3-QTL pyramiding lines (3QLs) and 4-QTL pyramiding lines (4QLs) developed previously. Three new pyramiding lines, 2QL-1/*gs3*, 3QL-3/*gs3* and 4QL-3/*gs3*, were developed in the HJX74 genetic background (**Supplementary Figure 3**). In the same way, *gs3* and *gw8* genes were introduced into the pyramiding lines carrying multiple SER-QTLs, 2QLs, 3QLs and 4QLs developed previously. Three new pyramiding lines, 2QL-5/*gs3*/*gw8*, 3QL-10/*gs3*/*gw8* and 4QL-1/*gs3*/*gw8*, were developed in the HJX74 genetic background (**Supplementary Figure 4**).

**Figure 5:**
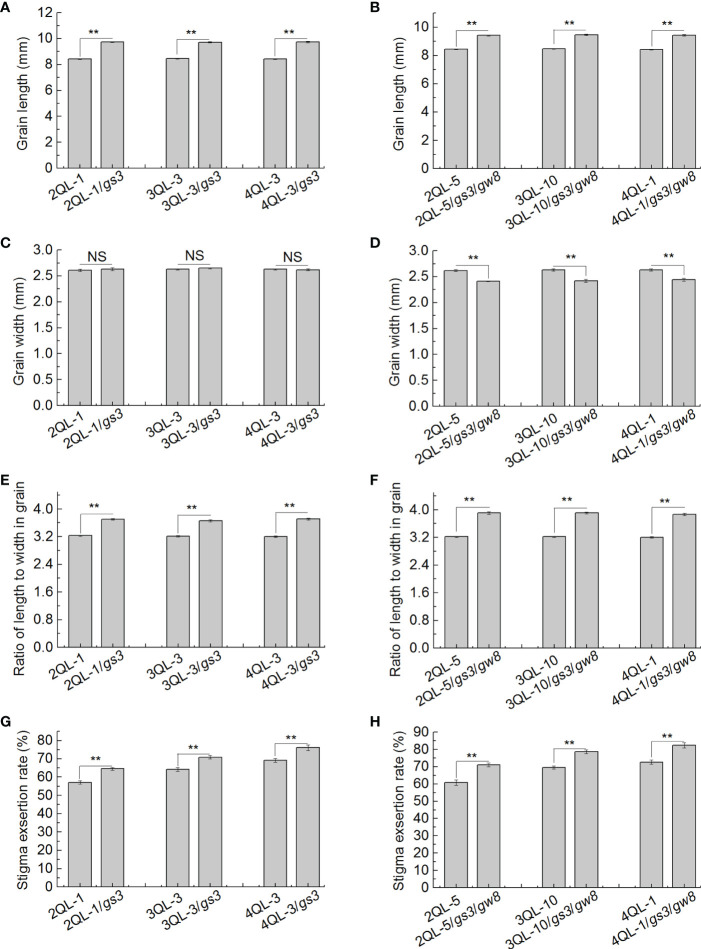
Grain shape and SER in pairs of pyramiding lines. Grain length **(A, B)**, grain width **(C, D)**, grain RLW **(E, F)**, and SER **(G, H)** in pairs of pyramiding lines with and without grain shape gene *gs3* and pyramiding lines with and without grain shape genes *gs3* and *gw8*. **P ≤ 0.01, Student’s *t* test. SER, stigma exsertion rate. RLW, ratio of length to width.

Compared with the pyramiding lines without *gs3*, the grain lengths of the pyramiding lines with *gs3*, 2QL-1/*gs3*, 3QL-3/*gs3* and 4QL-3/*gs3*, were significantly longer, but the grain width had no significant difference. The grains of pyramiding lines with *gs3* and *gw8*, 2QL-5/*gs3*/*gw8*, 3QL-10/*gs3*/*gw8* and 4QL-1/*gs3*/*gw8*, were significantly longer and narrower than those without *gs3* and *gw8* (**Figures 5A–D**). In grain RLW, the pyramiding lines with *gs3* and *gs3*/*gw8* were larger than those without *gs3* and *gs3*/*gw8*, while the pyramiding lines with *gs3*/*gw8* were larger than those with only *gs3* (**Figures 5E, F**).

SERs of three pairs of pyramiding lines with and without *gs3* and three pairs of pyramiding lines with and without *gs3* and *gw8* were compared in pairs. In three pairs of pyramiding lines with and without *gs3*, SERs of 2QL-1, 3QL-3 and 4QL-3 were 57.0%, 64.2% and 69.0% respectively, while those of 2QL-1/*gs3*, 3QL-3/*gs3* and 4QL-3/*gs3* increased to 64.5%, 70.8% and 76.2% respectively. The SERs of pyramiding lines with *gs3* were significantly higher than those of the pyramiding lines without *gs3* (**Figure 5G**). In three pairs of pyramiding lines with and without *gs3* and *gw8*, SERs of 2QL-5, 3QL-10 and 4QL-1 were 60.7%, 69.5% and 72.6% respectively, while those of 2QL-5/*gs3*/*gw8*, 3QL-10/*gs3*/*gw8*, 4QL-1/*gs3*/*gw8* increased to 71.1%, 78.7% and 82.4% respectively. SERs of pyramiding lines with *gs3* and *gw8* were significantly higher than those of the pyramiding lines without *gs3* and *gw8* (**Figure 5H**). These results indicated that the effect of grain shape on SER occurred not only in low SER background, but also in high SER background.

### Correlation between grain shape and stigma shape

To reveal the relationship between grain shape and stigma shape, stigma length and stigma width were measured in HJX74, 4 SSSLs, SSSL-*gs3*, SSSL-*gw5*, SSSL-*GW7* and SSSL-*gw8*, and 11 pyramiding lines, PL-*gs3*/*gw5*, PL-*gs3*/*GW7*, PL-*gs3*/*gw8*, PL-*gw5*/*GW7*, PL-*gw5*/*gw8*, PL-*GW7*/*gw8*, PL-*gs3*/*gw5*/*GW7*, PL-*gs3*/*gw5*/*gw8*, PL-*gs3*/*GW7*/*gw8*, PL-*gw5*/*GW7*/*gw8* and PL-*gs3*/*gw5*/*GW7*/*gw8*. Stigma RLWs were then calculated by the values of stigma length and stigma width in each line.

**Figure 6:**
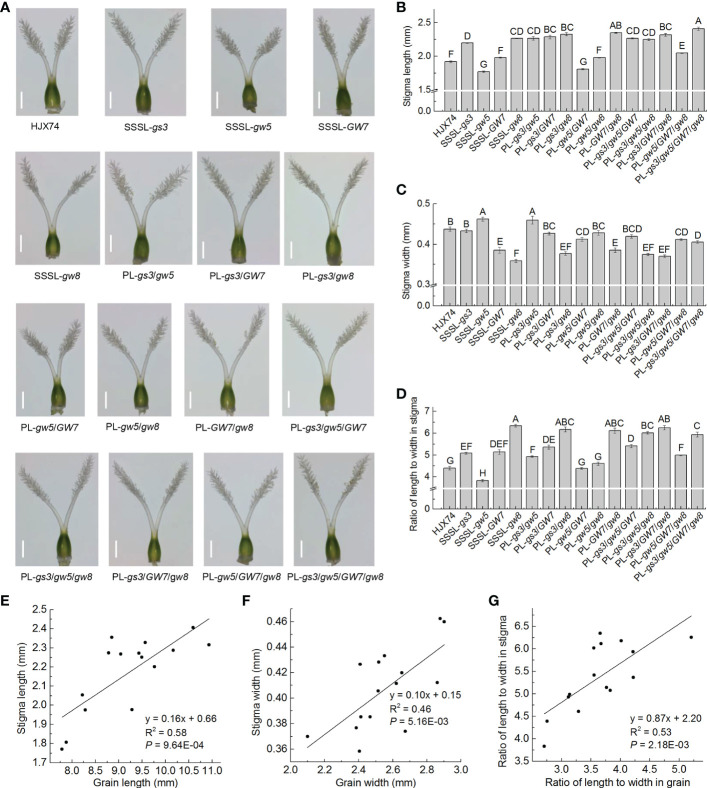
Stigma shape and its correlations with grain shape. **(A)**, Stigma shape of the SSSLs and PLs. Scale bar, 0.5 mm. **(B-D)**, Stigma length **(B)**, stigma width **(C)**, and stigma RLW **(D)** in the SSSLs and PLs. Data are shown as mean ± S.E. of two cropping seasons. Capital letters indicate significant differences at the 0.01 level. **(E-G)**, Correlations between grain length and stigma length **(E)**, between grain width and stigma width **(F)**, and between grain RLW and stigma RLW **(G)**. The average value of each line in two cropping seasons is used to perform correlation analysis. R^2^ represents the percentage of x contribution to y phenotype variation. SSSL, single-segment substitution line. PL, pyramiding line. RLW, ratio of length to width.

In stigma lengths, HJX74 was 1.92 mm, while 4 SSSLs were 1.77 mm to 2.27 mm, and 11 pyramiding lines were 1.81 mm to 2.41 mm (**Figures 6A, B** and **Supplementary Table 9**). In grain lengths, HJX74 was 8.38 mm, while 4 SSSLs were 7.79 mm to 9.77 mm, and 11 pyramiding lines were 7.88 mm to 10.93 mm (**Figures 3C**, **4A**; **Supplementary Tables 6**, **8**). Regression correlation between stigma length and grain length was a positive with the coefficient of determination (R^2^) of 0.58 (**Figure 6E**). In stigma widths, HJX74 was 0.44 mm, while 4 SSSLs were 0.36 mm to 0.46 mm, and 11 pyramiding lines were 0.37 mm to 0.46 mm (**Figures 6A, C** and **Supplementary Table 9**). In grain widths, HJX74 was 2.55 mm, while 4 SSSLs were 2.40 mm to 2.88 mm, and 11 pyramiding lines were 2.10 mm to 2.90 mm (**Figures 3D**, **4B**; **Supplementary Tables 6**, **8**). Regression correlation between stigma width and grain width was positive with R^2^ value of 0.46 (**Figure 6F**). In stigma RLWs, HJX74 was 4.40 mm, while 4 SSSLs were 3.83 mm to 6.35 mm, and 11 pyramiding lines were 4.39 mm to 6.26 mm (**Figures 6A, D** and **Supplementary Table 9**). In grain RLWs, HJX74 was 3.29 mm, while 4 SSSLs were 2.71 mm to 3.83 mm, and 11 pyramiding lines were 2.75 mm to 5.21 mm (**Figures 3E**, **4C**; **Supplementary Tables 6**, **8**). The stigma RLW was positively correlated with the grain RLW, and the R^2^ value was 0.53 (**Figure 6G**).

Taken together, stigma shape, including stigma length, stigma width and stigma RLW, was positively correlated with grain shape. The R^2^ values between stigma length and grain length, stigma width and grain width, and stigma RLW and grain RLW were close to 0.5. These results demonstrate that the genes *gs3*, *gw5*, *GW7* and *gw8* controlling grain shape have pleiotropic effects on stigma shape, and the stigma shape is partially affected by grain shape in rice.

### Correlation between grain shape and SER

**Figure 7:**
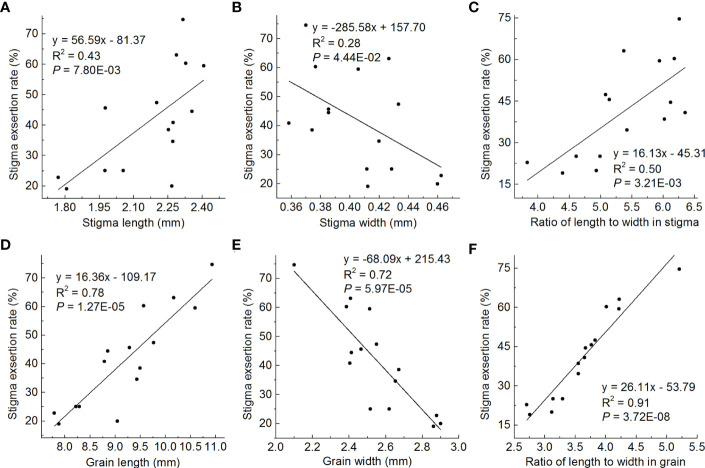
Regression correlations between stigma shape and SER, and grain shape and SER. **(A-C)**, Correlations between stigma length and SER **(A)**, stigma width and SER **(B)**, and stigma RLW and SER **(C)**. **(D-F)**, Correlations between grain length and SER **(D)**, grain width and SER **(E)**, and grain RLW and SER **(F)**. The average value of each line in two cropping seasons is used to perform statistical analysis. R^2^ represents the percentage of x contribution to y phenotype variation. SER, stigma exsertion rate. RLW, ratio of length to width.

Regression correlation analysis was used to detect the relationships between stigma shape and SER, and grain shape and SER in the lines containing different genotypes of grain shape in the HJX74 genetic background. In stigma shape, stigma length was positively correlated with SER (R^2^ = 0.43) (**Figure 7A**), stigma width was negatively correlated with SER (R^2^ = 0.28) (**Figure 7B**), and stigma RLW was positively correlated with SER (R^2^ = 0.50) (**Figure 7C**). In grain shape, grain length was positively correlated with SER (R^2^ = 0.78) (**Figure 7D**), grain width was negatively correlated with SER (R^2^ = 0.72) (**Figure 7E**), and grain RLW was positively correlated with SER (R^2^ = 0.91) (**Figure 7F**). These results showed that the effect of grain shape on SER was greater than that of stigma shape. In grain shape, the grain RLW had a greater effect on SER than grain length and grain width. Therefore, the grain RLW was the most important factor affecting SER.

## Discussion

### The grain shape has a partial effect on SER

In Asian cultivated rice, *indica* and *japonica* are two subspecies, in which *indica* tended to has slender grain and *japonica* mostly has a short and wide grain (Zhang et al., 2021). It is well known that the SER of *indica* rice is generally higher than that of *japonica* rice, which leads to generally obtain higher hybrid seed yield using *indica* MSLs than using *japonica* MSLs (Zhou et al., 2017; Dang et al., 2020; Jiang et al., 2021). It was found that the *GS3* gene controlling grain length had a positive effect on SER (Miyata et al., 2007; Li et al., 2014; Zhou et al., 2017; Xu et al., 2019; Liu et al., 2022) and *GW5* and *GW2* genes controlling grain width had a negative effect on SER (Zhou et al., 2017). However, the pleiotropic effect of the genes controlling grain shape were not always obvious (Zhou et al., 2017; Xu et al., 2019). In this study, we found that although grain length and grain width had significant correlated with SER, the grain RLW had the greatest effect on SER, with R^2^ value as high as 0.91 (**Figures 7D–F**). This finding can explain the fact that the SER of *japonica* rice is generally lower than that of *indica* rice. On the other hand, dozens of QTLs responsible for the SER have been identified in rice genome, many of them are independent of grain shape (Marathi and Jena, 2015; Liu et al., 2019; Tan et al., 2020; Tan et al., 2021; Tan et al., 2022a). Therefore, SER is only partially affected by the grain shape.

### Relationships of stigma shape with grain shape and SER

Stigma is a part of spikelet, located above ovary, surrounded by palea and lemma. At flowering, palea and lemma open, and the BSP of stigma may extend outside the glume. After flowering, palea and lemma are closed, and the BSP of stigma may exsert outside the glume (Khumto et al., 2018). Therefore, the stigma shape should be closely related to the grain shape and SER (Dang et al., 2016; Zhou et al., 2017). Wild rice has long and large stigma and strong outcrossing ability (Marathi et al., 2015). During the domestication process of cultivated rice, the stigma trait and natural outcrossing ability have already degenerated (Parmar et al., 1979; Marathi et al., 2015). Only few QTLs for stigma size including stigma length and stigma width were mapped. Some QTLs controlled stigma size and SER (Jiang et al., 2021), while others only controlled stigma size and didn’t influence SER (Uga et al., 2003; Yan et al., 2009; Zhou et al., 2017). In this study, we found that the genes controlling grain shape had pleiotropic effects on stigma shape. Correlation analysis showed that stigma length, stigma width and stigma RLW were weakly positively correlated with grain length, grain width and grain RLW respectively, with R^2^ values of about 0.50 (**Figures 6E–G**). Similarly, stigma length, stigma width and stigma RLW were also weakly correlated with SER, with R^2^ values of 0.50 or less (**Figures 7A–C**). These results showed that stigma shape was partly affected by grain shape, and has a partial effect on SER. The influence of grain shape on SER was partly caused by the influence on stigma shape. Recently, cellular examination and transcriptomic analyses revealed that three grain shape regulatory genes, *GS3*, *GW8* and *GS9*, cooperatively regulate cell division during pistil development (Zhu et al., 2023). This may be the molecular mechanism of the pleiotropy of grain shape regulatory genes on stigma shape and SER.

### The strategy for improving SER in rice

Cultivated rice lost the outcrossing ability during domestication (Parmar et al., 1979; Marathi et al., 2015). MSLs of hybrid rice need to restore the outcrossing ability to meet the needs of hybrid seed production (Taillebois et al., 2017). The stigma exsertion of rice breaks through the closure of palea and lemma, prolongs the pollination period, compensates for the asynchronous flowering time of male and female parents, and thus improves the outcrossing ability of MSLs (Rahman et al., 2017a; Zou et al., 2020). Therefore, improving SER is the key goal of MSL breeding. In recent decades, a large number of QTLs for SER have been located in rice genome, laying a foundation for improving SER (Marathi and Jena, 2015; Rahman et al., 2017b; Guo et al., 2022; Liu et al., 2022). Recently, eighteen QTLs for SER from *O*. *sativa*, *O*. *glaberrima*, and *O*. *glumaepatula* were mapped in the SSSLs of the HJX74 genetic background (Tan et al., 2020; Tan et al., 2021; Tan et al., 2022a). Eleven of the QTLs were used to develop pyramiding lines in the HJX74 genetic background. The pyramiding lines carrying 5-6 QTLs showed as high SER as wild rice. The phenotypic analysis showed that the increase of SER did not lead to the change of grain shape in most pyramiding lines. The results indicated that the high-SER trait can be reconstructed by pyramiding of the QTLs in rice (Tan et al., 2022b). In this study, we found that grain shape is another factor affecting rice SER, which affects SER by partially affecting stigma shape (**Figures 6** and **7**). Grain shape regulatory genes had pleiotropic effect on SER, and genes controlling long grain or narrow grain, such as *gs3*, *GW7* and *gw8*, could improve SER (**Figure 3**). The pyramiding line PL-*gs3*/*GW7*/*gw8* showed the largest grain RLW and the highest SER (**Figure 4**), which will be a fine breeding resource. Our results demonstrate that long grain rice is beneficial to the reconstruction of high SER trait. Therefore, the MSLs with high SER can be developed by pyramiding of the QTLs for SER and the genes controlling slender grain. Our finding implies that the breeding of MSLs with high SER is more difficult in *japonica* rice with short and wide grain than in *indica* rice with slender grain.

## Data availability statement

The original contributions presented in the study are included in the article/**Supplementary Material**. Further inquiries can be directed to the corresponding authors.

## Author contributions

GZ and SW designed the experiments and supervised the research works. QT performed most of the experiments. SC, ZG, QL, ZY, GC, SL, WY, JZ, YB, SB, and ZL performed a part of experiments. HZ and GL participated in material development. GZ and QT analyzed the data and wrote the manuscript. All authors contributed to the article and approved the submitted version.
