# Supplementary material for: Grain shape is a factor affecting the stigma exsertion rate in rice
Source: Front Plant Sci. 2023 Jan 31;14:1087285. doi: 10.3389/fpls.2023.1087285 (PMC9927237; doi:10.3389/fpls.2023.1087285)
Supplement: Supplementary file 1 [file DataSheet_1.docx]

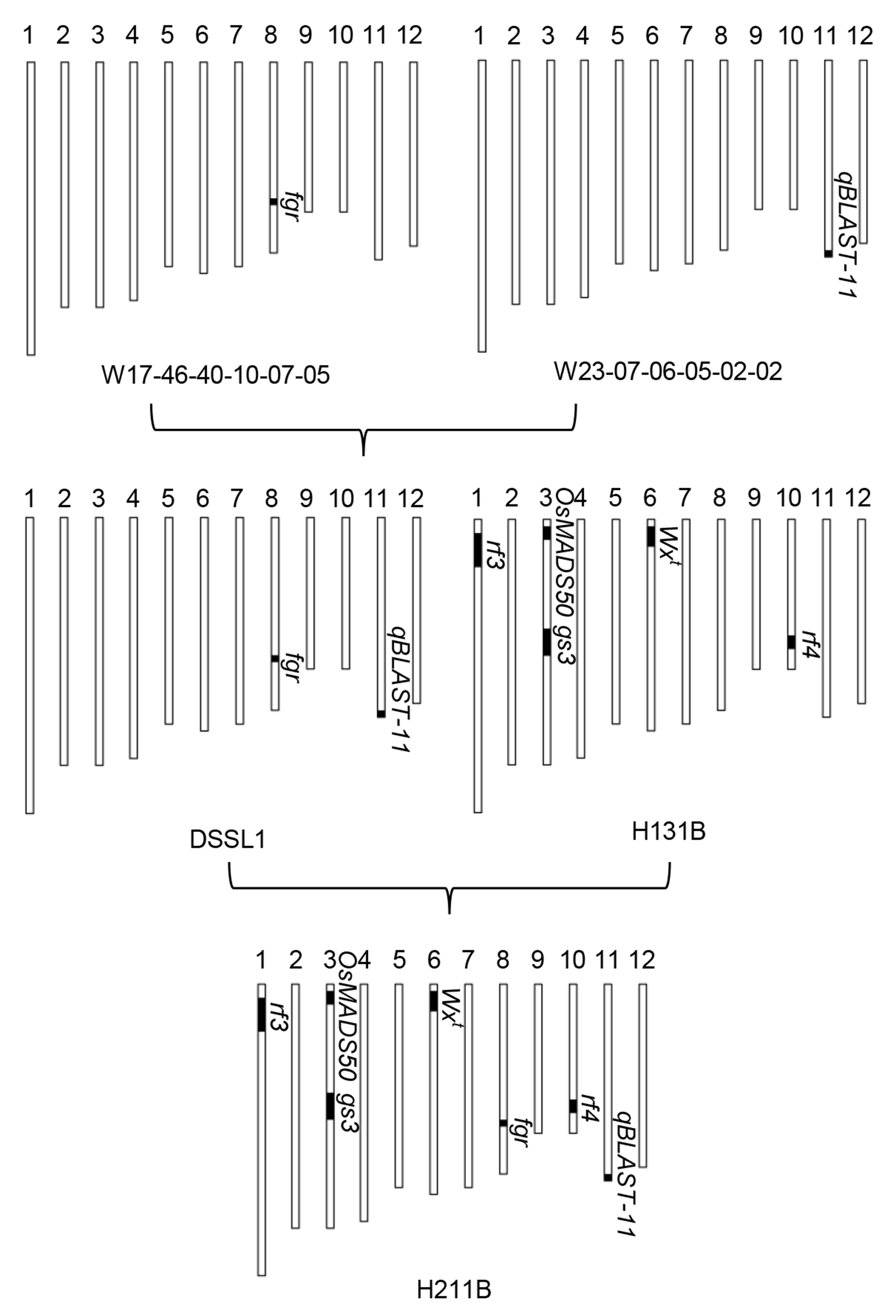


**Supplementary Figure 1** Development of the maintainer line H211B. The vertical bars are the graphical chromosomes. Black regions represent the substitution segments with target genes, and white regions represent the HJX74 genetic background.


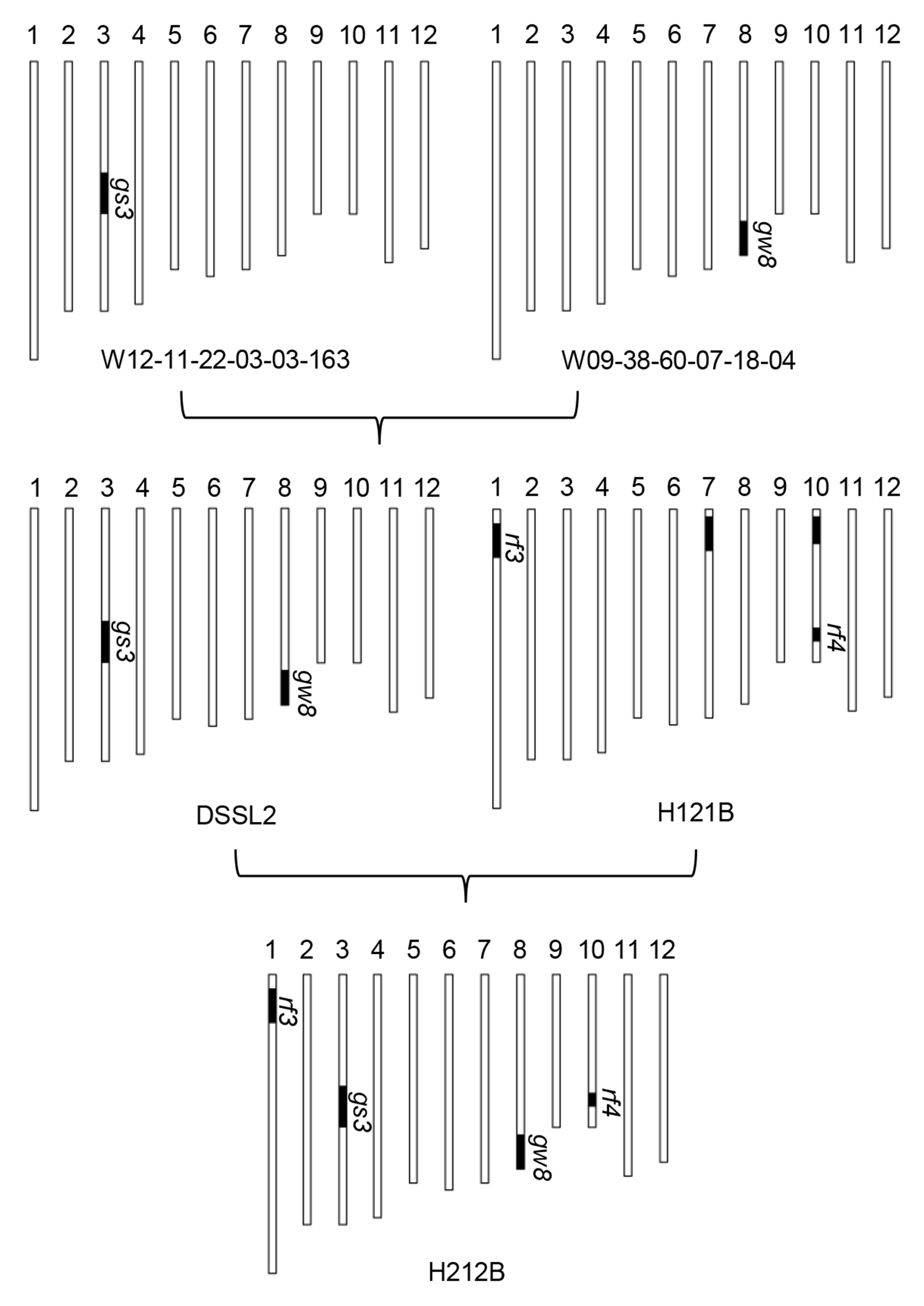


**Supplementary Figure 2** Development of the maintainer line H212B. The vertical bars are the graphical chromosomes. Black regions represent the substitution segments with target genes, and white regions represent the HJX74 genetic background.


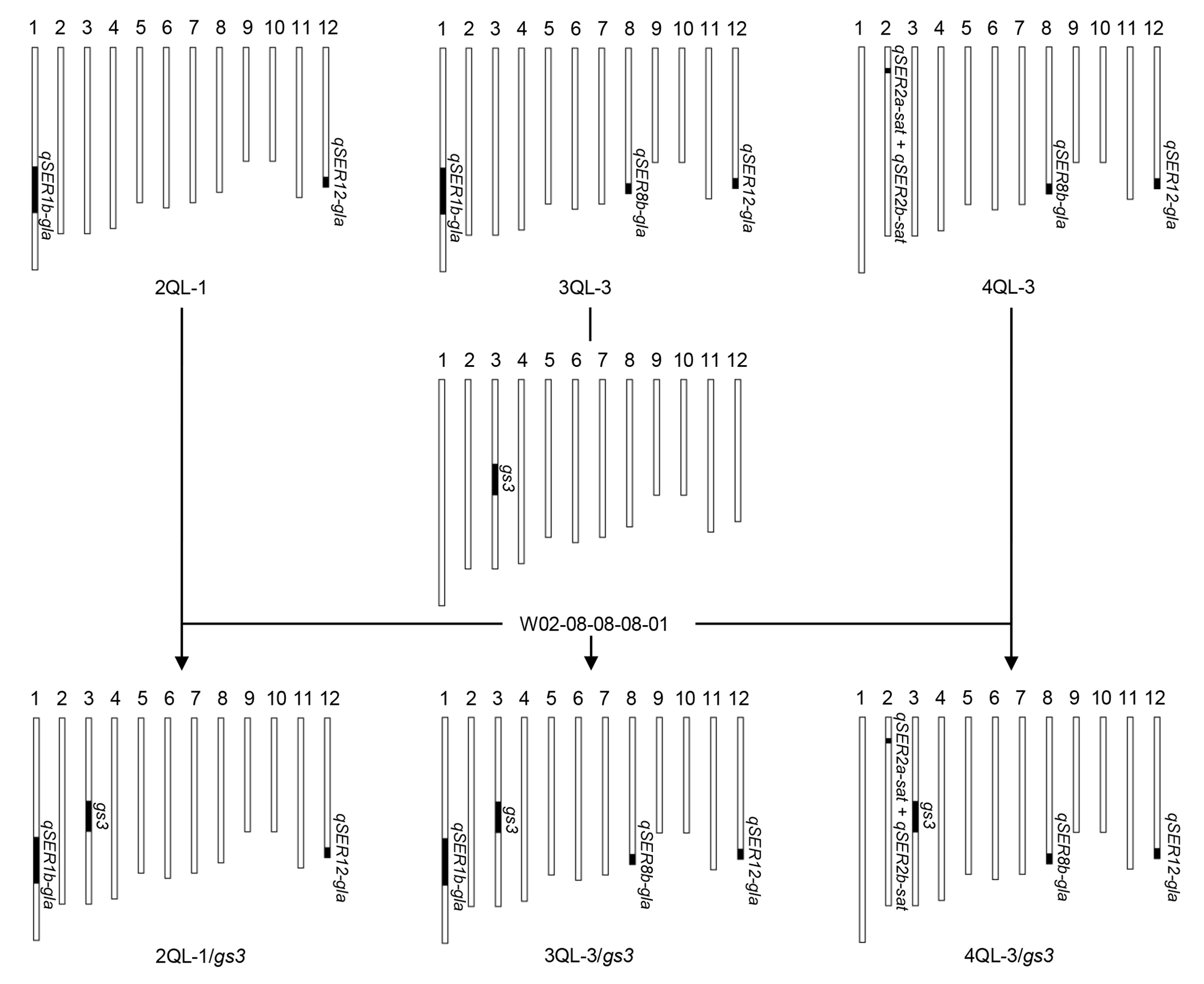


**Supplementary Figure 3** Development of the pyramiding lines combining SER-QTLs and the grain shape gene *gs3*. The vertical bars represent graphical chromosomes. Black regions represent the substitution segments with target genes, and white regions represent the HJX74 genetic background.


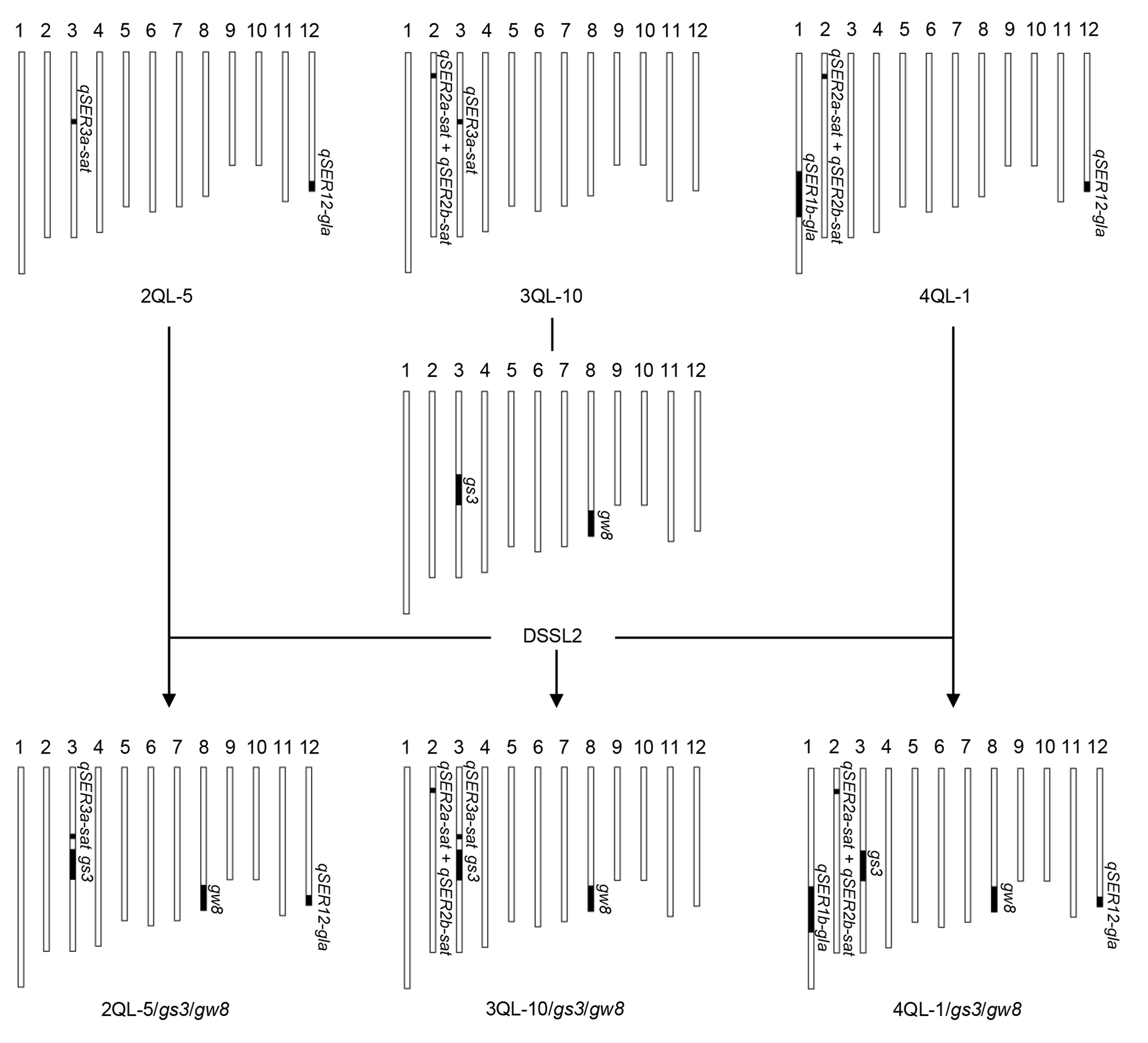


**Supplementary Figure 4** Development of the pyramiding lines combining SER-QTLs and grain shape genes *gs3* and *gw8*. The vertical bars are the graphical chromosomes. Black regions represent the substitution segments with target genes, and white regions represent the HJX74 genetic background.
